# Puzzling out the ecological niche construction for nitrogen fixers in a coastal upwelling system

**DOI:** 10.1093/ismeco/ycaf018

**Published:** 2025-02-04

**Authors:** Marcos Fontela, Daniel Fernández-Román, Esperanza Broullón, Hanna Farnelid, Ana Fernández-Carrera, Emilio Marañón, Sandra Martínez-García, Tamara Rodríguez-Ramos, Marta M Varela, Beatriz Mouriño-Carballido

**Affiliations:** Instituto de Investigacións Mariñas (IIM-CSIC), Vigo, Spain; Centro de Investigación Mariña da Universidade de Vigo (CIM-UVIGO), Vigo, Spain; Center of Marine Sciences (CCMAR), Universidade do Algarve, 8005-139 Faro, Portugal; Centro de Investigación Mariña da Universidade de Vigo (CIM-UVIGO), Vigo, Spain; Ocean and Earth Science, National Oceanography Centre, University of Southampton, Southampton, United Kingdom; Department of Biology and Environmental Science, Centre for Ecology and Evolution in Microbial Model Systems (EEMiS), Linnaeus University, Kalmar, Sweden; Instituto de Oceanografía y Cambio Global, Universidad de Las Palmas de Gran Canaria (ULPGC), Las Palmas 35214, Spain; Centro de Investigación Mariña da Universidade de Vigo (CIM-UVIGO), Vigo, Spain; Centro de Investigación Mariña da Universidade de Vigo (CIM-UVIGO), Vigo, Spain; Centro Nacional Instituto Español de Oceanografía, (IEO-CSIC), Centro Oceanográfico de A Coruña, Paseo Marítimo Alcalde Francisco Vázquez, n° 10, A Coruña 15001, Spain; Centro Nacional Instituto Español de Oceanografía, (IEO-CSIC), Centro Oceanográfico de A Coruña, Paseo Marítimo Alcalde Francisco Vázquez, n° 10, A Coruña 15001, Spain; Centro de Investigación Mariña da Universidade de Vigo (CIM-UVIGO), Vigo, Spain

**Keywords:** biological nitrogen fixation, ecological niche, upwelling bays, NW Iberia upwelling, nitrogen limitation

## Abstract

Diazotrophs are a diverse group of microorganisms that can fertilize the ocean through biological nitrogen fixation (BNF). Due to the high energetic cost of this process, diazotrophy in nitrogen-replete regions remains enigmatic. We use multidisciplinary observations to propose a novel framework for the ecological niche construction of nitrogen fixers in the upwelling region off NW Iberia—one of the most productive coastal regions in Europe—characterized by weak and intermittent wind-driven upwelling and the presence of bays. The main diazotroph detected (UCYN-A2) was more abundant and active during summer and early autumn, coinciding with relatively high temperatures (>16°C), low nitrogen:phosphorus ratios (N:P < 7.2), and a large contribution of ammonium (>75%) to the total dissolved inorganic nitrogen available. Furthermore, nutrient amendment experiments showed that BNF is detectable when phytoplankton biomass and productivity are nitrogen limited. Seasonally recurrent biogeochemical processes driven by hydrography create an ecological niche for nitrogen fixers in this system. During the spring–summer upwelling, nondiazotroph autotrophs consume nitrate and produce organic matter inside the bays. Thereafter, the combined effect of intense remineralization on the shelf and sustained positive circulation within the bays in late summer–early autumn, conveys enhanced ammonium content and excess phosphate into the warm surface layer. The low N:P ratio confers a competitive advantage to diazotrophs since they are not restricted by nitrogen supply. The new nitrogen supply mediated by BNF could extend the productivity period, and may be a key reason why upwelling bays are more productive than upwelled offshore waters.

## Introduction

Nitrogen (N) is a key element in the biosphere that limits the growth of primary producers in marine and land ecosystems [1]. Despite being very abundant in the atmosphere, it is only available for a reduced group of organisms, termed diazotrophs, able to reduce atmospheric dinitrogen (N_2_) gas into biologically assimilable forms, through biological dinitrogen fixation (BNF). The traditional focus on archetypal marine N_2_-fixers [filamentous cyanobacteria and diatom–diazotroph associations (DDAs)] has been expanded to include unicellular cyanobacteria and multiple groups of heterotrophic Bacteria and Archaea [2]. While the BNF activity of some of these groups is still uncertain or biogeochemically insignificant [3], the small diazotrophic cyanobacteria *Candidatus* Atelocyanobacterium thalassa (hereafter UCYN-A) has emerged as a key player in the marine nitrogen cycle [4], showing characteristics of a N_2_-fixing organelle, or “*nitroplast*” [5].

Despite decades of intense research, the control factors of diazotrophy remain enigmatic. It was traditionally assumed that the ability to access the vast atmospheric N_2_ pool gives an ecological advantage to diazotrophs where and when fixed N concentrations are low, whereas BNF would be irrelevant at enhanced availability of combined N resources [2]. However, energetically, fixing N_2_ is only marginally (~25%) more costly than using nitrate [6], and recent studies have expanded diazotroph global distribution to N-enriched regions [7–10], including subpolar and polar waters [11–14], coastal zones [15–17], upwelling ecosystems [18–20], and even the aphotic ocean [21].

Methodological approaches to investigate the relevance of control factors on diazotrophy include modeling studies [22, 23], nutrient addition experiments in the lab or with natural populations [6, 24, 25], and database analyses to explore relationships between environmental variables and the presence or activity of diazotrophs [10]. However, deciphering the independent effect of environmental properties on the distribution and activity of diazotrophs is not trivial: first, because environmental conditions are correlated to each other, and second, because nutrient concentration does not necessarily inform about nutrient availability, since low concentrations can be the result of phytoplankton consumption [26]. Few studies have investigated the drivers of diazotrophy in N-enriched regions, revealing correlations with temperature, chlorophyll-a (*Chla*), and inorganic nutrient content [9, 18, 27]. Whether these relationships are circumstantial or the result of control mechanisms for diazotrophy remains enigmatic.

The coastal upwelling region off the western Iberian Peninsula marks the northern limit of the Canary Current Upwelling Ecosystem, characterized by coastal embayments known as Rías [28]. In this area, upwelling is weak and intermittent, and the Rías are considered an extension of the shelf [29]. The predominance of along-shore northeasterly winds in spring/summer causes seasonal upwelling and positive circulation—surface waters from the Rías move toward the ocean, while deeper, nutrient-rich oceanic waters flow into the Rías [30, 31]. These nutrient inputs, enhanced by remineralization within the Rías and on the shelf [32], drive, among other factors, high phytoplankton production, which supports one of the most important blue economies based on living resources in Europe [33]. During early spring, when nutrient availability is high, the phytoplanktonic community is dominated by large diatoms and autotrophic nanoflagellates [34], while smaller diatoms and heterotrophs coexist during summer, as regenerated nutrients become more significant [35]. From October to March, prevailing southerly winds promote downwelling events [30, 31] and negative circulation—where surface waters flow into the Rías while deeper waters are expelled—associated with low phytoplankton growth conditions [36].

Knowledge about diazotrophy in this region is limited to a few observations. Relatively low BNF rates, mainly attributed to UCYN-A, were reported on the shelf off Ría de Vigo in the summer of 2009 [37, 38]. Ten samplings carried out between February 2014 and December 2015 at the shelf off Ría de A Coruña confirmed relatively low BNF rates in the region, which were higher (up to 0.095 nmol N L^−1^d^−1^) in surface waters during summer upwelling and relaxation [20]. Under these conditions, the diazotroph community was dominated by UCYN-A2, whereas with downwelling noncyanobacterial diazotrophs [i.e. heterotrophic Bacteria and Archaea, hereafter noncyanobacterial diazotroph (NCD)] were dominant [19]. Despite these insights, the factors driving BNF in this system remain unknown, as the limited temporal resolution of previous studies does not allow to reconstruct the environmental conditions accompanying the variability in diazotrophic composition and activity. Here, using a large data set of multidisciplinary observations, we describe in detail the ecological niche construction of nitrogen fixers in the upwelling region off NW Iberia.

## Materials and methods

### Sampling and hydrography

The dataset comprises 79 1-day sampling events within a period of ~4.5 years (2014–2018, Table S1). All samples belong to four different locations (Fig. S1). There were 10 samplings from the northern limit of the upwelling system, taken in the adjacent shelf off Ría de A Coruña (43.42° N, 8.44° W, 80 m depth) between February 2014 and December 2015 (NICANOR [20]). Next, there were 55 samplings with almost weekly resolution at a central station in the inner Ría de Vigo (42.24° N 8.78° W, 40 m depth) between March 2017 and April 2018 (REMEDIOS-seasonal [39]). Finally, there were 14 samplings over 2 weeks of summer 2018 with daily resolution (REMEDIOS-cruise [40]). Among them, 10 samples were taken inside the Ría de Pontevedra (42.36°N 8.78°W, 30 m depth) and 4 from the outer shelf (42.30°N 9°W, ~19.6 km apart, 100 m depth). During each sampling, profiles of temperature, salinity, and fluorescence were acquired with an SBE25plus CTD (SeaBird Electronics). Surface samples (1–3 m) were collected to determine dissolved inorganic nutrients (ammonium NH_4_^+^, nitrite NO_2_^−^, nitrate NO_3_^−^, phosphate PO_4_^3−^), *Chla*, primary production (PP), BNF, and DNA and RNA samples.

Samples for the determination of dissolved inorganic nutrients and total *Chla* were collected and frozen at −20°C [41, 42]. The fluorescence emitted by the *Chla* was measured from pigments extracted in 90% acetone at 4°C overnight using the spectrofluorometric method [20], and a Turner designs Trilogy fluorometer [43]. Seawater samples were spiked with 2–10 μCi of NaH14CO_3_ and incubated for 2–3 h starting at noon (REMEDIOS and nutrient addition experiments) or 24 h (NICANOR) in refrigerated incubators simulating the corresponding *in situ* irradiance. More detailed methodological procedures along with comprehensive hydrographic descriptions for each of the sites and conditions can be found in [20, 39, 40]. Since biological samples were restricted to the surface, the environmental parameters included as factors in subsequent statistical analysis are the median value of the first 5 m of the water column. Inorganic nutrient information has also been interpreted in terms of the N:P ratio, the relationship between total dissolved inorganic nitrogen (DIN) and phosphate:


$$ \mathrm{N}:\mathrm{P}\ \mathrm{ratio}=\mathrm{DIN}/{{\mathrm{PO}}_4}^{3-}=\left({{\mathrm{NO}}_3}^{-}+{{\mathrm{NO}}_2}^{-}+{{\mathrm{NH}}_4}^{+}\right)/{{\mathrm{PO}}_4}^{3-} $$

An N:P ratio = 16 denotes the fulfillment of Redfield stoichiometry [44]. Deviations from this ratio provide insights into the nutrient availability: N:P > 16 denotes excess nitrogen over phosphorus availability, whereas N:P < 16 indicates potential nitrogen limitation and excess phosphate. The fraction of NH_4_^+^ in total DIN [%NH_4_^+^ = NH_4_^+^ /(NO_3_^−^ + NO_2_^−^ + NH_4_^+^)], expressed as a percentage, was the variable informing about nitrogen speciation.

### Biological nitrogen fixation rates

Estimates of BNF activity at surface (1–2 m) are available for 34 sampling dates (43% of total samplings). BNF rates were determined with the ^15^N_2_ bubble addition technique [45]. Triplicate 2-L acid-cleaned polycarbonate bottles (Nalgene) were sealed with silicone septa caps and 3 ml of ^15^N_2_ (98 atom%, Cambridge Isotope Laboratories, Lot #I-16727 and #I-19168) were injected with a gas-tight syringe. The bottles were gently mixed and incubated for 24 h simulating *in situ* conditions of temperature and light with running-surface water and neutral-mesh shading to mimic surface irradiance. After incubation, each sample was filtered through precombusted (4 h, 450°C) 25 mm Whatman GF/F filters, (0.7 μm nominal pore size) using low vacuum (<100 mmHg), and filters were stored frozen (−20°C). A time-zero bottle was also filtered to calculate the initial natural abundance of N isotopes in the particulate material. Before analysis, filters were thawed, dried (60°C, 24–48 h), and pelletized in tin capsules. Particulate organic nitrogen and carbon (PON and POC) content, as well as the relative abundance of stable nitrogen isotopes (^15^N/^14^N), was determined with a continuous-flow isotope-ratio mass spectrometer MAT253 (Thermo Finnigan) coupled to an elemental analyzer EA1108 (Carlo Erba Instruments) through a Conflo III interface (Thermo Finnigan). During analysis, a set of international reference materials were analyzed for δ15N calibration (USGS 40, USGS41a USGS-25, IAEA-N-1, and IAEA-N-2). An analytical measurement error of ±0.15‰ was calculated for δ15 N; the error estimate was obtained from replicate assays of the laboratory standard acetanilide interspersed between sample analyses. Detection limits and error propagation were evaluated (Table S2) [46], and the detection range spans from 0.04 to 0.26 nmol·L^−1^d^−1^ with a mean of 0.10 ± 0.05 nmol·L^−1^d^−1^. BNF rates reported for this upwelling ecosystem are likely underestimated due to methodological issues [47] and should be viewed as conservative.

### Nutrient addition experiments with natural planktonic communities

Four nutrient addition experiments were performed (coinciding with different climatological seasons of 2017) along with the *in situ* sampling of natural planktonic communities at the inner part of Ría de Vigo (Fig. S1). The experimental design was performed in triplicates (three Whirl-pak® bags filled with 5.4 L of sample per treatment) and included a control (no additions performed) and two addition treatments: (i) NO_3_^−^ treatment, amended with 10–15 μM NO_3_^−^ and (ii) NH_4_^+^ treatment, amended with 10–15 μM NH_4_^+^. Besides, both addition treatments included a mix of organic nutrients (5 μM glucose and 5 μM amino acids) in all experiments and 1 μM of phosphate (PO_4_^3−^) in winter and spring experiments. A detailed description of the experimental setup is available in Text S1. Experiments lasted 3 days, and samples were taken every 24 h to monitor changes in *Chla* and PP. Samples for RNA and BNF rates were collected after 24-h incubation. The magnitude of the responses was estimated as response ratios (RRs) between the value of the variable at 24 h in the amendment treatment (AT) and the control (C), AT/C.

### Bulk DNA sampling, extraction, and nested amplification of the *nifH* gene and Illumina sequencing

For 71 sampling dates (90% of total) surface water was sampled for DNA collection. Between 7.5 and 10 L of seawater per replicate (4 replicates) were collected and filtered using silicone tubes and peristaltic pumps through sterile STERIVEX 0.22 μm pore size filters (Millipore, USA). Summer samples of July 2018 were prefiltered with a 200 μm mesh filter. Filters were preserved with 1.8 ml of lysis buffer (50 mM Tris-HCl pH 8.3, 40 mM ethylenediaminetetraacetic acid (EDTA) pH 8.0, 0.75 M sucrose), immersed in liquid nitrogen and subsequently stored at −80°C until further analysis. DNA was extracted using the PowerWater® DNA Isolation Kit (Mobio, Carlsbad, CA, USA), quantified and quality-checked (according to the A260/A280 ratio) using a spectrophotometer NanoDrop 2000TM (Thermo Fisher Scientific). Amplification of *nifH* gene and sequencing of the amplicons were performed following [48, 49]. The polymerase chain reactions (PCR) were run on a T100TM Thermal cycler (BIO-RAD) [19]. The amplified products were purified using PCR Extract Mini Kit (5PRIME) and quantified using a spectrophotometer NanoDrop 2000™ (Thermo Fisher Scientific). Prior to sequencing, an additional PCR amplification of 10 cycles with custom barcoded *nifH1* and *nifH2* primers was carried out. After purification and library preparation from the barcoded PCR products, paired-end sequencing was performed using the MiSeq® reagent kit with V2 chemistry (500 cycles) at the facilities of IMGM Laboratories GmbH (Germany) on the Illumina MiSeq® Next Generation Sequencing technology (Illumina Inc.).

### RNA extraction, cDNA generation, and nifH gene quantification

For 15 sampling dates (19% of total), surface water was sampled for RNA collection following the same procedure described for DNA. Total RNA extraction from Sterivex® filters was performed by using 200 μm low-binding zirconium beads (OPS diagnostics) and the QIAGEN RNeasy Mini Kit (Cat.no 74104) and RNase-Free DNase Set (Cat.no 79254). DNase treatment was done with AMBION Turbo DNA free Kit (Invitrogen, Cat.no: AM1907). First-strand complementary DNA (cDNA) synthesis was done with SuperScript® III First-Strand Synthesis System for RT-PCR, and total RNA concentration was quantified by Nanodrop & Qubit. To quantify *nifH* genes and expression of key diazotrophs identified in the amplicon libraries, 78 and 15 samples were amplified and quantified by quantitative PCR (qPCR) and reverse transcription qPCR (RT-qPCR), respectively, using Taqman primer probe sets (PrimeTime qPCR Assays, Integrated DNA Technologies) (see details in [20]). Briefly, the qPCR assays targeted the *nifH* gene of three common diazotrophic strains, namely, UCYN-A2 [50], UCYN-A1 [51], and γ-proteobacterium affiliated phylotype γ-24774A11 [52] (Table S3). The PrimeTime® Gene Expression Master Mix (IDT), DNA/cDNA template, and primers-probe set (0.5 and 0.25 μM final concentrations, respectively) were combined with PCR-grade water (Sigma-Aldrich) to create a final reaction volume of 20 μl. Thermal cycling conditions for the qPCR assay consisted of a 3-min incubation at 95°C, followed by 45 cycles of 15 s at 95°C and 1 min at 60°C. Each qPCR run included nine 10-fold serial dilutions of standards that contained the targeted *nifH* fragments (gBlocks® Gene Fragments, IDT), and samples were run in triplicate, while standards were run in duplicate. Reactions were performed in a MyiQ2™ Real-Time PCR Detection System (Bio-Rad Laboratories). Amplification efficiencies were always greater than 90%. We performed an inhibition test by several dilutions at certain random samples, and we concluded that our samples were not inhibited. The limit of detection (LOD) was 9 *nifH* copies L^−1^, and the detected but not quantified (DNQ) limit was 87 *nifH* copies L^−1^ for UCYN-A2 and UCYN-A1, and 86 *nifH* copies L^−1^ for Gammaproteobacteria. Abundances below LOD were assigned 0 *nifH* copies L^−1^, whereas measurements higher than the LOD but less than the DNQ were assigned 1 *nifH* copies L^−1^.

### Bioinformatics and phylogenetic

Paired-end reads were merged, selected, and quality-filtered; the chimera was removed; and amplicon sequence variants (ASVs) were determined with the *dada2* pipeline [53]. Sequences with stop codons or with frameshift errors were excluded from the analysis. Non-*nifH* sequences were filtered out against the database compiled in [54] using the Hidden Markov Model-based algorithm HMMER. The remaining sequences were aligned to a reference alignment, in the “genome879” *nifH* database (https://wwwzehr.pmc.ucsc.edu/Genome879/). The phylogenetic affiliation of the translated ASVs into the canonical *nifH* clusters defined by [55] was performed by BLASTx [56] using genome-derived sequences from the updated and curated *nifH* database [57] as references. Finally, ASVs were sorted according to their closest affiliation in the BLASTp database (>96% cutoff) [55]. Following BLASTp matching at the genus level, diazotrophs were categorized into nine taxonomical groups (Class level) for subsequent analytical purposes (Table S4). Rarefaction curves (rarefy function in vegan package [58]) showing a plateauing trend (Fig. S2) confirmed that DNA extraction, sequencing, and bioinformatics were appropriately implemented for most samples, and only 4 DNA samples out of 79 were discarded. We established that the diazotrophic community at each individual sample was dominated by Cyanobacteria when UCYN-A relative abundance met two criteria: (i) it was the most abundant ASV, and (ii) it represented individually more than a third (33%) of total relative abundance. Otherwise, we considered the community dominated by NCDs (Fig. S3).

**Figure 1:**
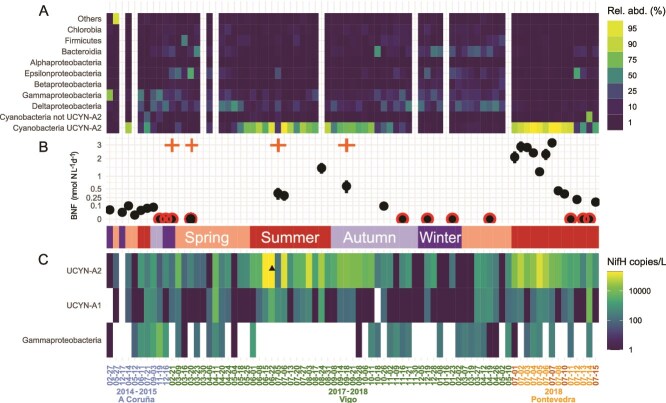
Diazotrophic abundance and community composition, and biological nitrogen fixation. Temporal variability of (A) *nifH* amplicon-based sequencing community composition (relative abundance %) for the most abundant taxa, ordered from top to bottom in increasing abundance. Blank gaps are discarded samples due to DNA extraction issues or unacceptable rarefaction curves. (B) Nitrogen fixation rate (nmol N L^−^1d^−1^) measurements. When values are below detection limits, they are represented with a red border. Error bars represent triplicate measurements (if not visible, they are not larger than the dots). Note the square root transformation on the *y*-axis (not linear to help in the visualization of small values). (C) Quantitative *nifH* gene copy abundance (qPCR, *nifH* gene copies L^−1^) data for UCYN-A2, UCYN-A1, and *Gammaproteobacteria* 24774A11. Note that the color scale is logarithmic. UCYN-A2 abundance on 22 June 2017 exceeded the scale’s range by an order of magnitude (1.7 ± 0.7 × 10^6^  *nifH* transcripts copies·L^−1^), and it was replaced by a black triangle for visualization purposes. The same figure without this visual solution can be seen in Fig. S9. Climatological season is represented in the inner colored band. The *x*-axis is shared for the three graphs, sampling dates (format: year/month/day) colored according to location (color code for the locations in Fig. S1). Times when nitrogen amendment experiments were performed are indicated with orange crosses in the upper margin of (B).

### Statistical analysis

All statistical analyses were done in R (v4.1.2; R Core Team 2021). The community composition of diazotrophs was investigated by principal coordinates analysis (PCoA, *vegan* package) with an ordination based on the Bray–Curtis dissimilarity distances matrix. The environmental parameters were fitted onto the PCoA ordination and represented as vector overlays when their contribution to the observed differences was relevant (*envfit* function in the *vegan* package, *P*-value <.001).

Then, we assessed the degree of niche overlap among diazotroph taxonomical groups with respect to those relevant environmental drivers identified from the PCoA (sea surface temperature, N:P ratio and %NH_4_^+^). Niche overlap analysis is based on nonparametric kernel density estimation [59, 60]. Based on a null model analysis, the difference in niche overlap between groups was considered statistically significant at a *P*-value <.01.

Finally, we investigated if those environmental drivers determined ASV’s relative abundance. The *corncob* method is based on beta-binomial count regression for correlated observations and is suited for modeling microbial abundances based on high-throughput sequencing data [61, 62]. For each individual ASV, a regression model of relative abundance versus environmental data tested the differential abundance across the existent N:P ratio, fraction of NH_4_^+^ in total DIN, and surface water temperature conditions, also controlling the effect of these environmental drivers on the dispersion. A false discovery rate of <0.5% was selected as threshold.

## Results

### Diazotrophic community composition and biological nitrogen fixation

A total of 7030 ASVs were identified based on *nifH* sequencing. The nine categorized taxonomical groups represented 97.6% of the total ASVs, with *Cyanobacteria* and *Proteobacteria* jointly being 81.6% (45% and 36.6%, respectively) (Fig. 1A). The community composition shows similar patterns between sampling sites. The relative abundance of cyanobacterial diazotrophs was large in summer/early autumn, while NCDs were more relevant in winter/spring, when *Cyanobacteria* were almost absent and the diazotrophic community was more diverse (Fig. S4). UCYN-A2 was the main UCYN-A ecotype and the most abundant ASV. *Deltaproteobacteria* was the second taxa in relative abundance, followed by *Gammaproteobacteria*. The temporal variability of relative abundance based on *nifH* sequencing was coherent with the pattern attained by absolute quantification by qPCR (Fig. 1A and C). Frequently, the quantification of UCYN-A2 was two orders of magnitude larger than UCYN-A1. *Gammaproteobacteria* was below the detection limit in more than half of the samples, and their mean absolute abundance was low (708 ± 1600 *nifH* gene copies L^−^1).

The mean BNF for all samples was 0.92 ± 1.1 nmol N L^−1^d^−1^ (*n* = 34), ranging from 0.025 nmol N L^−1^d^−1^ to 3.17 nmol N L^−1^d^−1^ (Fig. 1B), and it was below detection (i.e. zero) in 11 samples. BNF rates were higher during summer and early autumn in Ría de Vigo and Ría de Pontevedra (2017–2018), whereas they were in general lower in the northernmost location sampled at the shelf off Ría de A Coruña (2014–2015). Higher BNF rates coincided with relatively high *Cyanobacterial* diazotroph gene abundance, whereas NCDs dominated the diazotrophic community when BNF rates were undetectable (Fig. 1).

**Figure 2:**
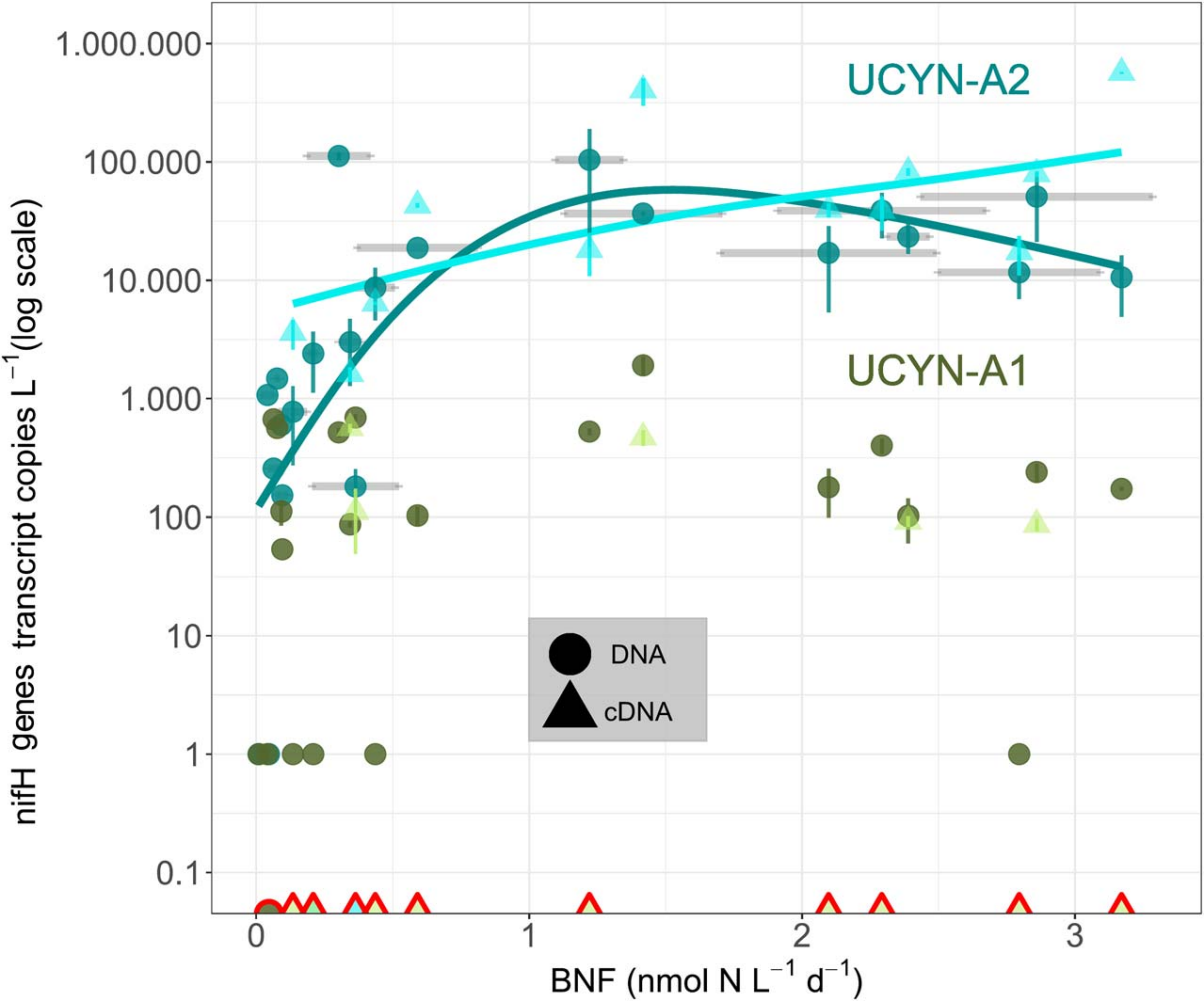
UCYN-A *nifH* gene abundance and expression. Relationship between BNF (nmol N L^−1^d^−1^) and the abundance of UCYN-A ecotypes (A1 and A2). Quantification of *nifH* gene copies/L by qPCR from DNA (circles) and *nifH* gene transcripts/L by RT-qPCR from cDNA (triangles). Note that the *y*-scale is logarithmic. Error bars in the *y*-axis represent the standard deviation from three replicates per sample (if not visible, they are not larger than the dots). Uncertainty in N_2_-fixation rates is represented in the *x*-axis with gray horizontal bars spanning the triplicate measurements (only for UCYN-A2). Samples with positive BNF and nondetection by qPCR are included on the *x*-axis with red bordering.

The log-transformed abundance of UCYN-A2 *nifH* transcripts (measured by qPCR based on cDNA) exhibited a positive relationship with BNF rates at rates lower than 1.5 nmol N L^−1^d^−1^ (GAM model, *P*-value = .0617, adjusted *R*^2^ = 0.55, deviance explained = 64%, Fig. 2). Above this BNF rate, a saturation relationship was observed. Transcription of *nifH* associated to UCYN-A2 also scaled linearly along BNF rates, while no relationship was found between BNF rates and UCYN-A1 *nifH* gene copies or transcripts (Fig. 2).

### Environmental control of diazotroph community structure

**Figure 3:**
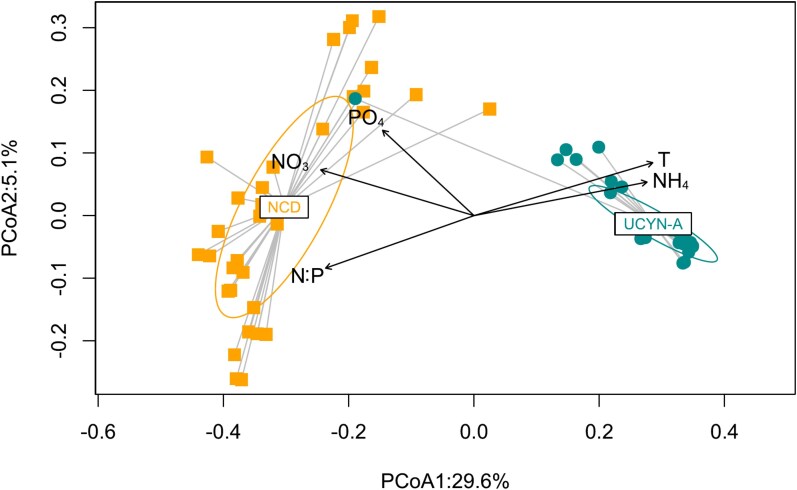
Taxa dominance is related to environmental conditions. PCoA based on the Bray–Curtis dissimilarity matrix illustrating the differences between samples dominated by UCYN-A (cyan circles) and NCDs (orange squares). Sample group centroids for each dominance situation and confidence ellipses displaying the standard deviation of centroid locations are also represented. The environmental factors (%NH_4_^+^, N:P ratio, *in situ* temperature, NO_3_^−^ and PO_4_^3−^) that contributed most to the community differences observed between these dominance situations were fitted through significant (*P* < .001) vector overlays (black color) onto the PCoA ordination. The first axis of the PCoA (*x*-axis) explains 29.4% of the variance and the second axis (*y*-axis) 5.1%.

PCoA analysis confirmed the existence of two groups of samples according to diazotroph community composition (Fig. 3). The first PCoA axis separated samples in which cyanobacterial diazotrophs dominate the community (cyan circles, UCYN-A) from those in which NCDs dominated the community (orange squares, NCDs). The lower dispersion observed in the UCYN-A group suggests a less diverse phylogenetic community. On the other hand, the larger dispersion of the NCD group [3] suggests higher phylogenetic diversity at the community level. This is confirmed by alpha diversity metrics (Fig. S5). The environmental parameters that most contributed to this community differences are *in situ* temperature, the fraction of NH_4_^+^ in total DIN (%NH_4_^+^), NO_3_^−^, N:P ratio, and PO_4_^3−^ (in that order, complete list of environmental variables and the model fitting coefficients available in Table S5). The N:P ratio, NO_3_^−^, and PO_4_^3−^ were negatively associated with temperature and %NH_4_^+^ in the ordination space. The dominance of UCYN-A in the community was associated with higher temperature and %NH_4_^+^ concurrent with a low N:P ratio.

**Figure 4:**
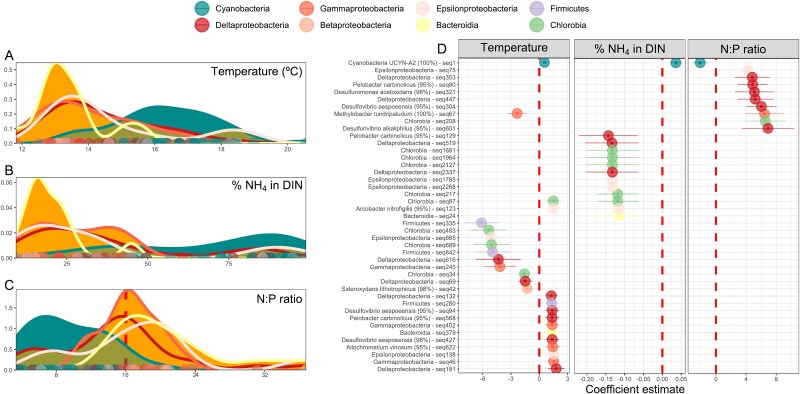
Temperature and inorganic nitrogen conditions are niche descriptors of the diazotrophic community. (A) Kernel density estimates for the taxonomical groups and the niche descriptors temperature [°C, (A)] and inorganic nutrient content, [% of NH_4_^+^ in total DIN, (B) and N:P ratio, (C)] and separated by cyanobacterial (cyan) and noncyanobacterial diazotrophs (orange). The red vertical line in (C) at N:P ratio = 16 delimits apparent nitrogen limitation conditions (when the ratio is <16, left side of the graph). Deviations from N:P ratio = 16 (red dashed line in Fig. 4C) separate conditions when there is phosphorus in excess (on the left side of the plot) from conditions of nitrogen surplus (on the right side). The *y*-axis represents the probability density function for the kernel density estimation. (D) Coefficients of models after beta-binomial regression for the environmental parameters temperature (left column) and inorganic nutrient content (% of NH_4_^+^ in total DIN, mid; N:P ratio, right column). Only significant models (*P* ≤ .05) from the whole diazotroph community (7030 ASVs) are shown. The coefficient estimate indicates positive or negative responses to the parameter and is shown with a 95% confidence interval. Specie identification in the *y*-axis label is only shown when the BLASTp result is ≥95% (percentage in parentheses). A detailed table with taxonomy info and complete *nifH* sequence for each one of these ASVs is available in Table S7. The color code is shared at the taxa level for both panels.

Kernel density estimates were used to assess the niche overlap of taxonomical groups in terms of temperature, %NH_4_^+^ in DIN, and N:P ratio (Fig. 4A–C, Table S6). *Cyanobacteria* peaked at 16°C, and they were present at the warmest temperature registered (19°C–20°C), whereas NCD groups like *Bacteroidia* and *Proteobacteria* (*Gamma*-, *Epsilon*-, and *Betaproteobacteria*) peaked around 13°C (Fig. 4A). The fraction of ammonium in total DIN was also a differential parameter for cyanobacteria (more present at higher NH_4_^+^ proportions, >75%) and NCDs (which showed higher abundance at low NH_4_^+^ proportions, 25%, Fig. 4B). Finally, *Cyanobacteria* occurred preferentially at low N:P ratio conditions (7.2), when there were potential N limitation and excess P, whereas NCD groups have their peak of occurrence at N:P ratios > 16 (Fig. 4C).

Additional statistical support at the ASV level with beta-binomial count regression models identified 25 ASV differentially abundant with regard to temperature, 13 ASVs with regard to the fraction of NH_4_^+^ in DIN, and 10 ASVs that are differentially abundant across the N:P ratio (out of the 7030 ASVs tested, Fig. 4D). Only UCYN-A2 has a significant differential abundance related to the three environmental drivers. UCYN-A2 was the only *Cyanobacteria* ASV with a temperature-mediated response, as well as the only ASV with a positive coefficient for the relevance of NH_4_^+^ in the total DIN, and a negative coefficient for the N:P ratio.

### Biological nitrogen fixation is driven by inorganic nitrogen availability

**Figure 5:**
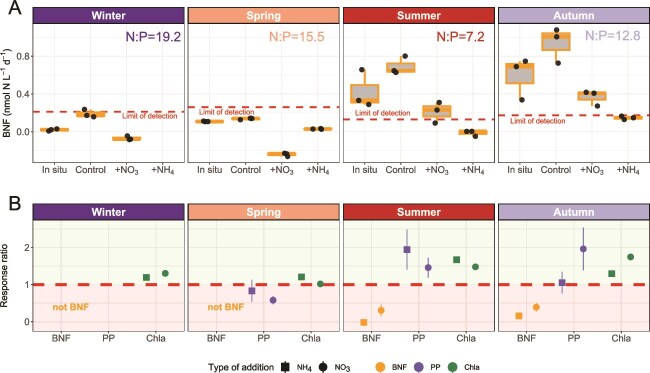
BNF decreases with inorganic nitrogen additions when the plankton community is N-limited. Response of BNF, primary production rates, and C*hla* after nutrient amendment microcosm experiments through each climatological season (*n* = 4). (A) BNF (nmol N L^−1^d^−1^) at *in situ* conditions (natural environment), inside the microcosm setting (“*Control*”) and the response after 24 h in the nitrate (+NO_3_^−^) or ammonia (+NH_4_^+^) amendment treatments. Red dashed horizontal line represents the methodological limit of detection for each triplicate of measurements (black dots). (B) Response ratio defined as the change in the absolute value of the variable with regard to control conditions after 24 h. Values below 1 (red dashed line) are mean reductions and values above 1 mean increases.

We performed controlled nitrogen amendment experiments to assess whether BNF activity occurs when the phytoplankton standing stock is N-limited, across four seasons (winter, spring, summer, and autumn) in the inner Ría de Vigo. As reported previously (Fig. 1), amendment experiments showed higher *in situ* (natural environment) BNF rates (0.025 nmol N L^−1^d^−1^ to 3.17 nmol N L^−1^d^−1^) in summer and autumn, whereas they were below the detection limit in winter and spring (Fig. 5A). A significant (ANOVA, *P*-value <.05, Fig. 5A) positive response of PP and *Chla* to nitrogen additions (i.e. phytoplankton standing stock was N-limited) was observed in summer and autumn experiments, coinciding with higher BNF in the field (Fig. 5B). In all seasons with measurable BNF activity, BNF rates were inhibited and significantly reduced when ammonium and nitrate were added, respectively (ANOVA, *P*-value <.05, Fig. 5A).

## Discussion

### UCYN-A2 is the main active N_2_-fixer

We revealed through alternative approaches that the abundance of diazotrophs shows seasonality in the NW Iberian upwelling system, with UCYN-A2 being most abundant and active during summer and early autumn. High abundances of *nifH* UCYN-A2 transcripts were concomitant with measurable BNF rates, supporting the link between the expression pattern of UCYN-A2 with BNF rates. This agrees with the current knowledge that UCYN-A2 is more relevant in coastal waters than UCYN-A1 [63, 64], and supports the recognition of UCYN-A2 as the main active diazotroph in temperate upwelling regions [65]. UCYN-A2 was also the dominant diazotroph, with comparable BNF rates to those in this study, peaking in summer (ca. 2–3 nmol N L^−1^d^−1^), in both North Atlantic coastal waters [10] and the Subarctic North Pacific [14]. However, there are at least three lines of evidence that point to an unknown additional group of diazotrophs fixing N in this system. First, the relationship between BNF magnitude and the abundance of UCYN-A reaches a plateau at rates above 1.5 nmol N L^−1^d^−1^. Second, at the controlled conditions of the experimental nutrient additions, there is no relationship between the change in BNF rates and the absolute abundance of nifH transcript copies L^−1^ quantified by qPCR for UCYN-A2 (Fig. S6). Lastly, when the quantified *nifH* copies L^−1^ are combined with published cell-specific N_2_ fixation rates for UCYN-A (~55 fmol N cell^−1^ d^−1^ [66]) and NCD (0.69 ± 1.57 fmol N cell^−1^ d^−1^ [67]), the calculated BNF magnitude is much lower than observed (Fig. S7). The combination of low cell-specific rates and/or low abundance makes the UCYN-A and NCD contribution insufficient to explain the observed BNF rate. Therefore, another key diazotroph player must be involved, being diatom–diazotroph associations (DDAs, [68]) a possible explanation. DDAs were not detected within our DNA/RNA sequencing. This absence might be surprising, as this coastal upwelling region is dominated by diatoms during the spring–summer productive season [36]. Several diatom genera that are hosts of diazotrophs (like *Hemiaulus* or *Rhizosolenia*) are commonly present [69, 70], including in our samples [71]. Although the nondetection of DDA N_2_-fixers activity aligns with previous size-fractionated incubations that attributed BNF rates on the shelf off Ría de A Coruña exclusively to small diazotrophs [20], it remains uncertain whether this absence of DDA contribution is due to a methodological bias in the DNA amplification technique [72] or represents a true absence of diazotroph symbionts. To disentangle the contribution of DDA versus UCYN-A in this region, a closer visual inspection of the host cells, a DDA-targeted nanoSIMS approach, and/or size-fractionated incubations should be implemented in the future.

### Low nitrogen content and high temperature are the environmental drivers

Our results point out temperature and low nitrogen content (evaluated through the N:P ratio) as the environmental parameters that drive diazotroph abundance, community composition and activity in this coastal upwelling system. The relative enrichment of phosphorus as compared to nitrogen confers a competitive advantage to diazotrophs since they are not restricted by the nitrogen supply. This outcome, confirmed at community, taxa, and ASV levels, is consistent with previous results. Temperature and N:P ratio have been identified as the main controlling factors of diazotroph abundance in the North Pacific Ocean [27]. Warm temperature (>16°C) and lower NO_3_^−^ content have been suggested as drivers of UCYN-A2 abundances in the California upwelling region [18]. Additionally, summer observations carried out in the temperate western North Atlantic revealed that diazotroph community composition and BNF correlated positively with *Chla* and P availability [9].

**Figure 6:**
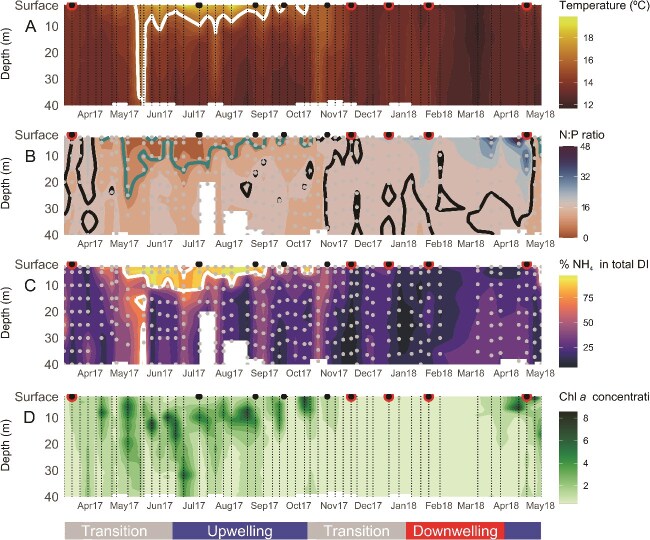
Hydrographic full-depth variability at weekly resolution at a central station in the inner Ría de Vigo during spring 2017–spring 2018. Time-series of the full-depth vertical distribution of (A) temperature (°C). The white solid line represents the isotherm of 16°C. (B) N:P ratio. The green and black solid line represents the isoline of 7.2 and 16, respectively. (C) % of NH_4_^+^ in DIN. The white solid line represents the isoline of 75%. (D) *Chla* concentration (mg·m^−3^). Dots represent sample vertical resolution. Surface black circles represent nitrogen fixation rate measurements. When values were below detection limits, they are represented with a red border. Climatological season is represented in the top-colored band. The hydrographic conditions with respect to upwelling (blue), downwelling (red), and transition (gray) in the low-colored band following [39].

In addition, the short-term increase in PP and *Chla* observed in our nutrient amendment experiments during summer and autumn, when a low N:P ratio exists (Fig. 5A), indicates that the phytoplankton standing stock was nitrogen-limited or at least responsive to nitrogen supply [73]. Interestingly, BNF rates were not completely suppressed after NO_3_^−^ addition in the nutrient amendment experiments. A possible explanation for this is that UCYN-A2 lives in symbiosis with prymnesiophyte algae (*Braaudosphera bigelowi*) exchanging fixed N for fixed carbon [64, 74]. Indeed, the genome of UCYN-A is so streamlined to fuel BNF that it even lacks genes for carbon fixation, oxygen-evolving photosystem II, or nitrate assimilation genes, so it is an obligate N_2_-fixer [75, 76] or even a new organelle [5]. Thus, UCYN-A symbiosis relies on N_2_ fixation even in N-rich environments [25]. It can be hypothesized that the BNF decreases after nitrogen addition in our microcosm experiments not because of inorganic nutrient inhibition, but due to the eukaryotic host algae being outcompeted by other phytoplankton. Groups such as diatoms, characterized by high maximum nutrient uptake and growth rates [77], may outcompete the slow-growing *Braaudosphera*/UCYN-A symbiosis after nutrient additions [78], resulting in decreased BNF.

### Puzzling out the diazotroph niche

Then, how is the diazotroph niche constructed in this coastal upwelling region? We propose a novel framework connecting hydrography and ecology through biogeochemical processes. The full-depth biogeochemical sequence and its connection with surface BNF is shown at a central station in the inner Ría de Vigo at weekly resolution for the period spring 2017–spring 2018 (Fig. 6). Starting with hydrography, a key feature of this upwelling system is the bidirectional exchange flow with a two-layer structure [79, 80]. During summer, when upwelling conditions prevail, a positive circulation occurs in the Rías: cold subsurface water enters through the lower layer (Fig. 6A), in contact with the bottom, while warm surface waters flow out toward the shelf [39]. The upwelling causes the uplifting of the relatively young Eastern North Atlantic Central Water (ENACW), which has an N:P ratio above or close to 16 (Fig. 6B, [29]) and a low ammonium content (NH_4_^+^ < 0.5 μmol kg^−1^, [81]).

**Figure 7:**
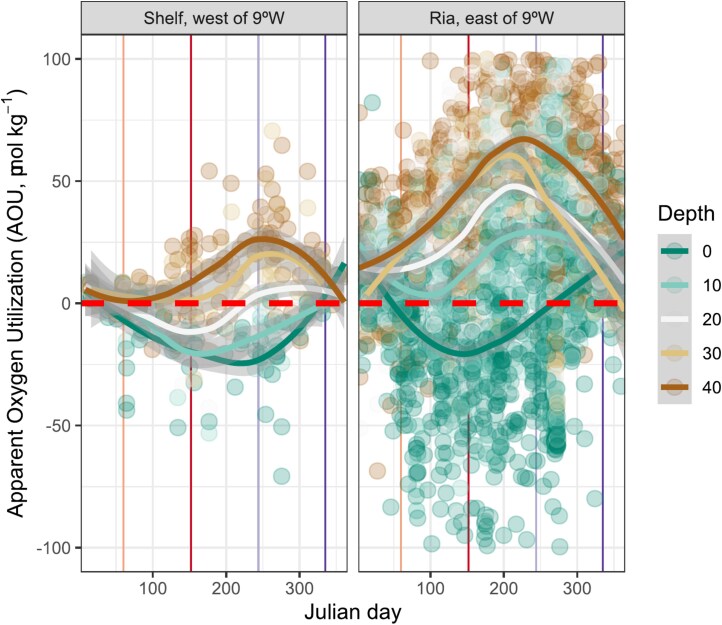
Annual variability of apparent oxygen utilization (AOU, μmol kg^−1^), a proxy of the biological production and consumption of oxygen, at the Ría de Vigo. It includes discrete samples of measured dissolved oxygen data from 1986 to 2018 [87] and the shelf/Ría separation criteria (panels left and right) were considered at 9°W longitude (westernmost location 9.5°W). Depth bands grouped each 10 m, from surface (0 m) to bottom (40 m, maximum depth of central station in the inner Ría de Vigo), have been modeled with a locally weighted polynomial regression (LOESS). Oversaturation of oxygen due to biological production (AOU <0, μmol kg^−1^) and undersaturation due to respiration (AOU > 0, μmol kg^−1^) are separated by the red dashed line. The vertical solid lines represent the onset of climatological seasons.

Following with ecology, this region shows a rapid phytoplankton response to upwelling pulses [40], as evidenced by the drawdown of inorganic nitrogen and the subsequent chlorophyll accumulation (Fig. 6B–D, Fig. S8). After nitrogen drawdown through phytoplankton uptake, a significant fraction of this fresh organic matter is remineralized within 1–2 weeks [81]. This recycling mechanism, also known as nutrient trapping, occurs when particulate organic material sinks out of the photic zone but remains within the upwelling system [81]. The signal of intense bottom remineralization that occurs over the shelf and within the Rías is conveyed into the inner part of the bays, where nutrient-trapping processes magnify and then uplift and exit in the surface outflow with the positive circulation [82]. The circulation outflow conveys a remineralization fingerprint that becomes more evident at the surface as the upwelling-favorable season proceeds (Fig. 6B and C). This remineralization fingerprint: (i) decreases the N:P ratio because P remineralization is faster than that of N [83–85] and (ii) accumulates ammonium regenerated within the ría via ammonification processes [86]. Evidence of cumulative remineralization is reflected in the seasonal evolution of apparent oxygen utilization (AOU), a proxy for the net biological production or consumption of oxygen, calculated as the difference between the observed dissolved oxygen and its saturation concentration (Fig. 7). When AOU at the Ría de Vigo is assessed with discrete samples of measured dissolved oxygen data from 1986 to 2018 [87], it shows lower negative values at the surface during spring and summer due to the oxygen production linked to the synthesis of organic matter by autotrophs. Later, AOU reaches its highest positive values in deeper layers as oxygen is consumed during the breakdown of this organic matter (Fig. 7).

**Figure 8:**
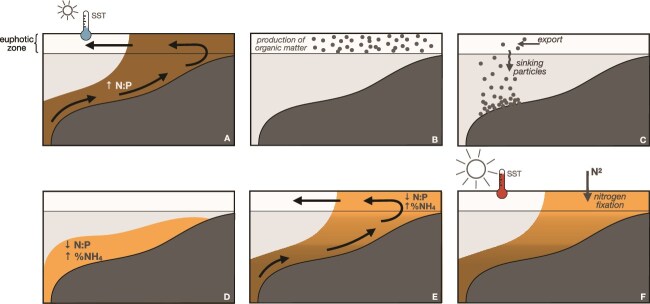
The sequential steps of the mechanistic biogeochemical niche construction process for nitrogen fixers. (A) At the beginning of the upwelling season, sea surface temperature (SST) is still cold. Upwelled ENACW brings nutrients upward, into the ría, sustaining an elevated N:P ratio and low content in the form of NH_4_^+^. (B) Phytoplankton grow within the surface layer due to the previous nutrient input and the necessary irradiance. Subsequently, (C) organic matter produced by phytoplankton is exported to the outer part of the ría, where it sinks. (D) This organic matter is remineralized near the bottom, where P content rapidly increases, lowering N:P levels. There, inorganic nitrogen is mostly in the form of NH_4_^+^. (E) Further into the upwelling season, upwelled waters are mixed with the remineralized P-rich waters, and positive upwelling circulation transports them toward the surface. (F) At the end of the summer upwelling season, SST has increased (>16°C) and nitrogen fixers can take advantage of it, in combination with the low N:P conditions.

We argue that in summer and early autumn, when BNF occurs, the signal from bottom remineralization processes—following the sustained organic matter production during the productive upwelling season—reaches the surface, conveyed by the positive circulation in the Rías (sequential steps schematized in Fig. 8). This creates the optimum niche for BNF in terms of temperature and inorganic nutrient content. The predominance of downwelling conditions in late autumn/winter, which promote negative circulation in the Rías, combined with low nutrient uptake by phytoplankton due to light limitation [29], and freshwater runoff events reset the inorganic nutrient content in the surface layer to nitrogen-replete conditions (Fig. 6) [88], thereby disrupting the diazotroph niche and preventing BNF from occurring. It is uncertain whether only an N:P ratio < 16 is important or the higher contribution of NH_4_^+^ over NO_3_^−^ to total inorganic nitrogen availability could also be a key factor in the formation of the diazotroph niche. Higher temporal and spatial resolution data covering different environmental conditions and/or multifactorial controlled experiments [89] are needed to unravel the specific role of temperature and the contribution of NH_4_^+^.

The conceptual framework proposed in our study resembles the mechanistic niche construction previously described in the eastern tropical North Atlantic Ocean [90], where nondiazotrophs facilitate BNF by creating an environment with excess phosphate. However, in our case, the trigger is the combination of predominant positive circulation within the bays during the upwelling season and the shorter turnover time of phosphorus compared to nitrogen during organic matter remineralization [84]. The specific characteristics of NW Iberia, such as the presence of elongated bays where upwelling is relatively weak and intermittent [29], enable the ecological niche construction for diazotrophy. Despite BNF representing a minor entry of new nitrogen into the euphotic zone compared to other physical processes [20], it may be critical in supporting phytoplankton growth at the end of the productive upwelling season. Therefore, these results could help explain why the upwelling bays are more productive compared to the upwelled offshore waters [32]. Future studies are needed to disentangle what are the key diazotroph players that extend the productive season of the upwelling bays, and the extent to which similar processes occur in other coastal upwelling regions.

## Supplementary Material

Fontela_SuppInfo_reviewed_ycaf018

Fontela_SuppInfoTableS1_ycaf018

Fontela_SuppInfoTableS2_ycaf018

Fontela_SuppInfoTableS7_ycaf018

## Data Availability

Source datasets supporting the current study are available in https://figshare.com/collections/Mixing_and_Phytoplankton_Growth_in_an_Upwelling_System/5604209 and https://data.mendeley.com/datasets/pm4r2pyyh3/2. *nifH* sequences are deposited in SRA with accession number PRJNA1184991 https://www.ncbi.nlm.nih.gov/bioproject/PRJNA1184991. Code and data to reproduce the results is publicly available in https://github.com/mfontela/nifH_niche
