# Supplementary material for: Puzzling out the ecological niche construction for nitrogen fixers in a coastal upwelling system
Source: ISME Commun. 2025 Feb 4;5(1):ycaf018. doi: 10.1093/ismeco/ycaf018 (PMC11931620; doi:10.1093/ismeco/ycaf018)
Supplement: Fontela_SuppInfo_reviewed_ycaf018 [file fontela_suppinfo_reviewed_ycaf018.pdf]

Supplementary information for

Puzzling out the ecological niche construction for nitrogen fixers in a coastal upwelling system

Marcos Fontela[1], Daniel Fernández Román[2], Esperanza Broullón[3], Hanna Farnelid[4], Ana Fernández Carrera[5], Emilio Marañón[2], Sandra Martínez Garcia[2], Tamara Rodríguez-Ramos[6], Marta Varela[6], Beatriz Mouriño[2]

Corresponding autor: mfontela@iim.csic.es

[1]Instituto de Investigacións Mariñas (IIM-CSIC), Vigo, Spain

[2]Centro de Investigación Mariña da Universidade de Vigo (CIM-UVIGO), Vigo, Spain

[3]Ocean and Earth Science, National Oceanography Centre, University of Southampton, Southampton, UK

[4]Department of Biology and Environmental Science, Centre for Ecology and Evolution in Microbial Model Systems (EEMiS), Linnaeus University, Kalmar, Sweden

[5] Instituto de Oceanografía y Cambio Global, Universidad de Las Palmas de Gran Canaria (ULPGC), 35214, Las Palmas, Spain.

[6]Centro Nacional Instituto Español de Oceanografía, (IEO-CSIC), Centro Oceanográfico de A Coruña, Paseo Marítimo Alcalde Francisco Vázquez 10, 15001, A Coruña, Spain.

Figure S1

Geographical location of the sampling stations. From North to South, shelf off Ría de A Coruña (43.42º N, 8.44º W, 80 m depth, blue dot), outer shelf of Ría de Pontevedra (42.30ºN 9ºW, 100 m depth, orange dot), inside Ría de Pontevedra (42.36ºN 8.78ºW, 30 m depth, yellow dot) and inner Ría de Vigo (42.24ºN 8.78ºW, 40 m depth, green dot).

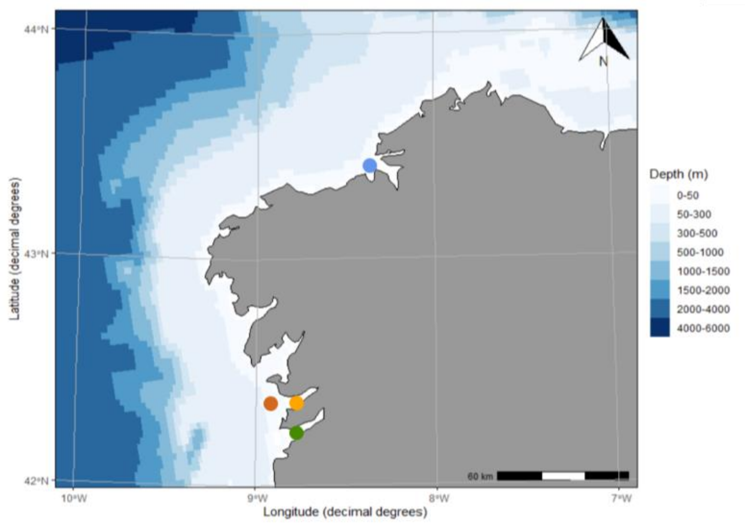

Figure S2

Rarefaction curves of *nifH* amplification. The plateau of the rarefaction curves suggests that the method captured most of the diazotrophic diversity in the samples.

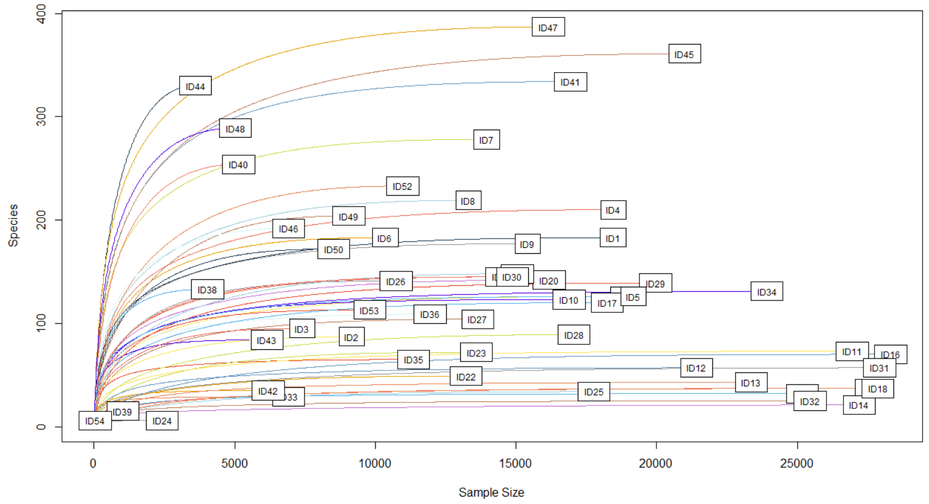

Figure S3

Relative abundance (%, in percentage) of the most dominant ASV by each sampling day. Points are color-coded according to the specific ASV that was most abundant on that day (legend). The UCYN-A group shows significantly higher abundances of the most dominant ASV compared to the NCD group, where the most abundant ASV tends to have lower relative abundances.

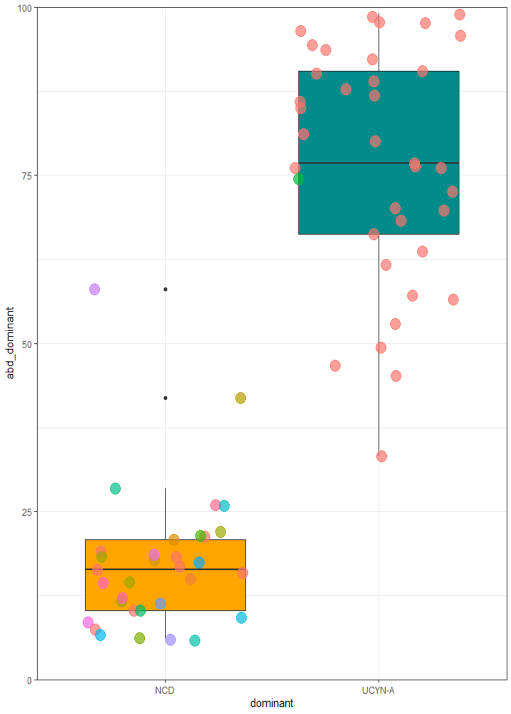

Figure S4

Relationship between diversity (Shannon index) and Cyanobacteria relative abundance (in percentage, %). The diazotrophic community is more diverse at low relative abundance of Cyanobacteria (UCYN-A)

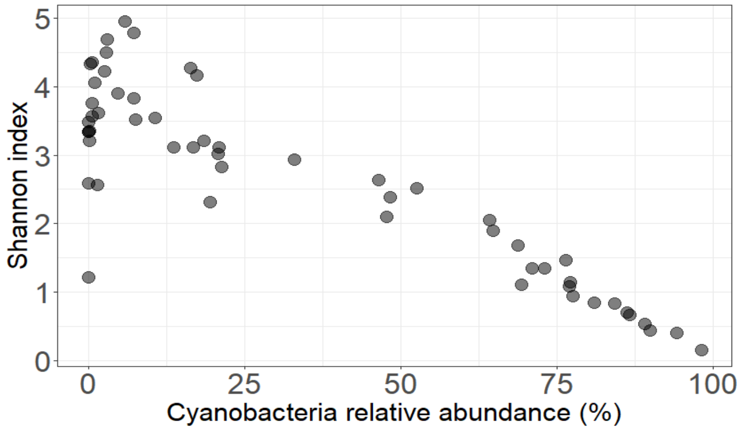

Figure S5

Alpha diversity (Shannon index) in relation to the dominant diazotrophic community.

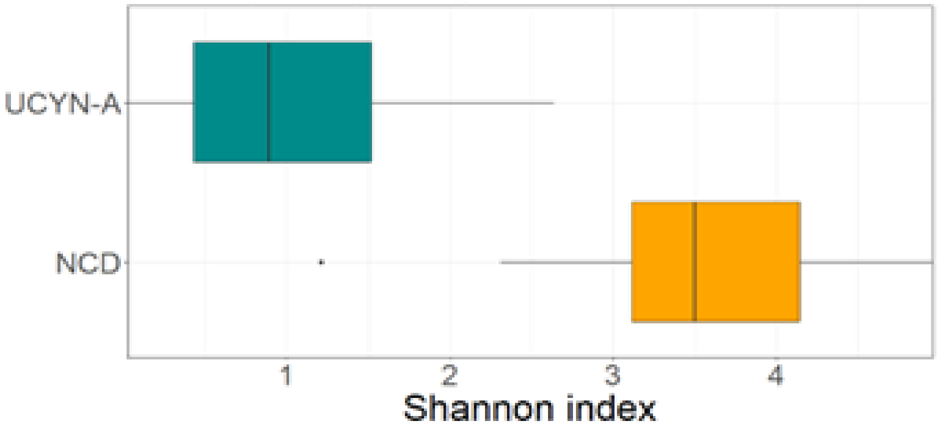

Figure S6

Null relationship between BNF (nmol N $L^{-1}d^{-1}$) measured rates and the quantitative *nifH* gene transcripts abundance (RT-qPCR, nifH transcripts copies L^{-1}) for UCYN-A2. Within the controlled conditions of the experimental additions, the change in BNF rates does not correlate with the absolute abundance identified using qPCR.

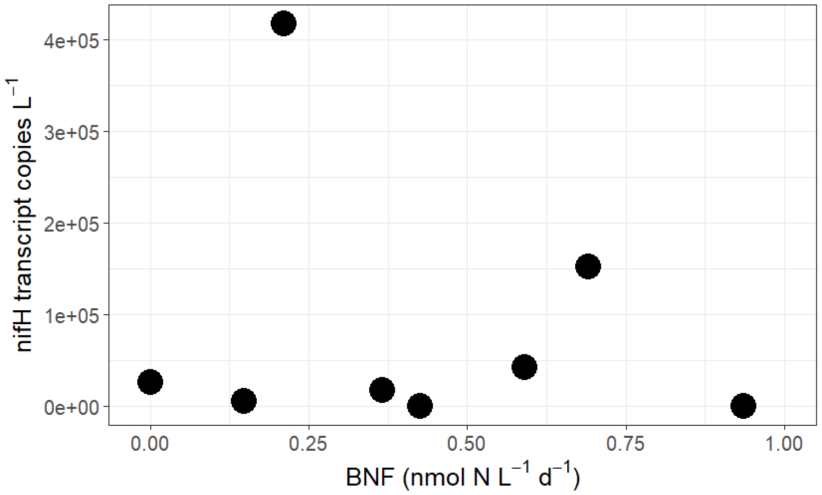

Figure S7

Observed versus expected BNF rates (nmol N $L^{-1}d^{-1}$). The expected BNF rates have been computed according to the quantified nifH copies L^{-1} combined with published cell-specific N_2 fixation rates for UCYN-A (~55 fmol N $cell^{-1}$ d^{-1},[1]) and NCD (0.69 ± 1.57 fmol N $cell^{-1}$ d^{-1},[2]). Additionally, the ratio of *nifH* gene copies to cell abundance for UCYN-A is 14±10[3] and it was considered as 1 for NCD as remain unknown[4]. Red dashed line is 1:1 relationship, note that the observed BNF magnitude (x-axis) is much higher than expected. Note that both axes are not linear (square transformation). Points are color-coded according to the *nifH* copies L^{-1} in logarithmic scale.

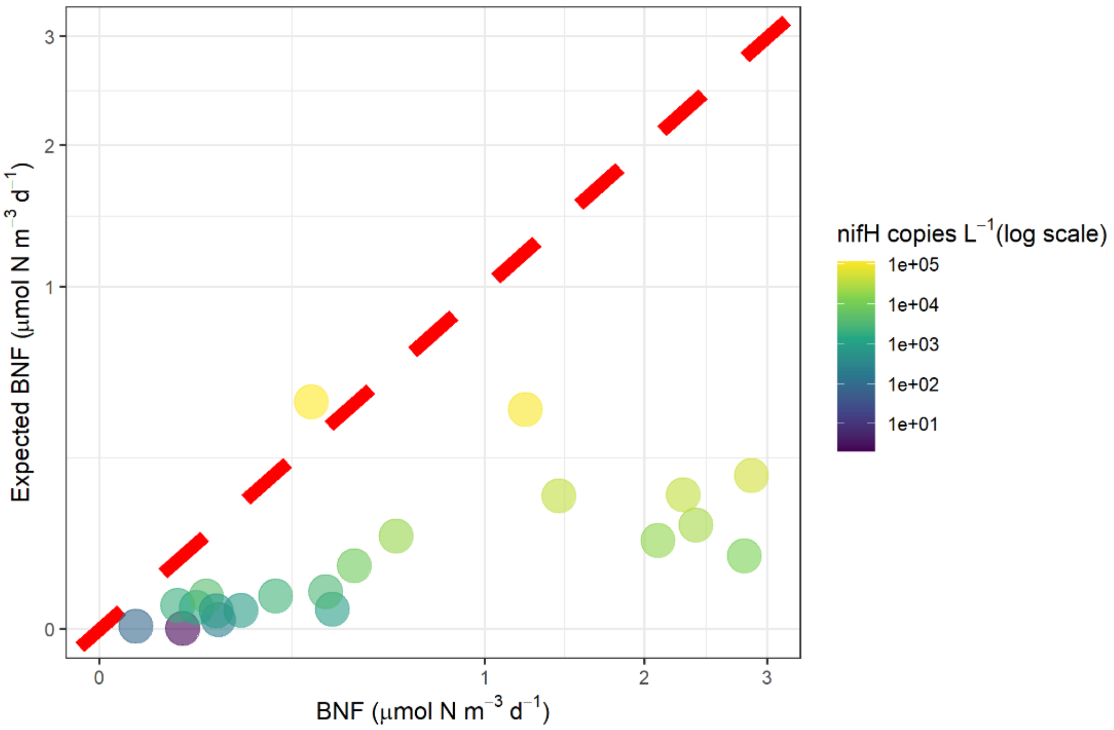

Figure S8

Time-series of the full-depth vertical distribution at weekly resolution at the central Ria de Vigo during spring 2017 – spring 2018 of total dissolved inorganic nitrogen (DIN, µmol kg^{-1}, upper plot) **and phosphate** (PO_4^{3-}, µmol kg^{-1}, lower plot). Dots represent sample vertical resolution. Surface black circles represents nitrogen fixation rate measurements. When values were below detection limits, they are represented with a red border. Climatological season is represented in the top colored band. The hydrographic conditions with respect to upwelling (blue), downwelling (red), and transition (gray) in the low colored band following[5].

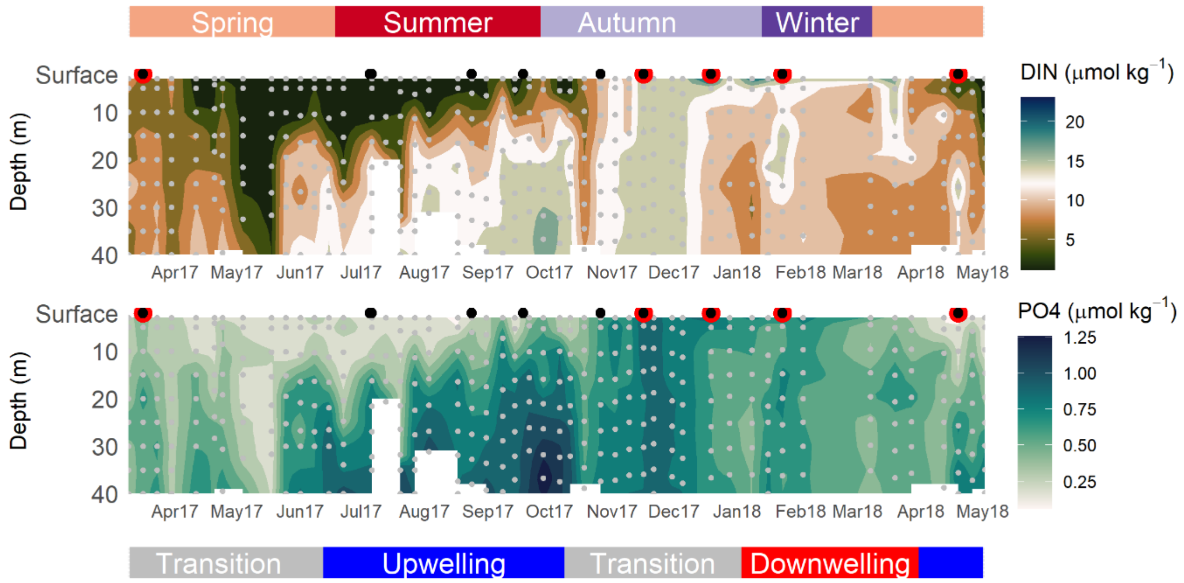

Figure S9

Quantitative *nifH* gene copy abundance (qPCR *nifH* gene copies L^{-1}), data for UCYN-A2, UCYN-A1 and *Gammaproteobacteria* 24774A11. Note that the color scale is logarithmic. Sampling dates in the x-axis (format: year/month/day) colored according to location (color code for the locations in Fig. S1).

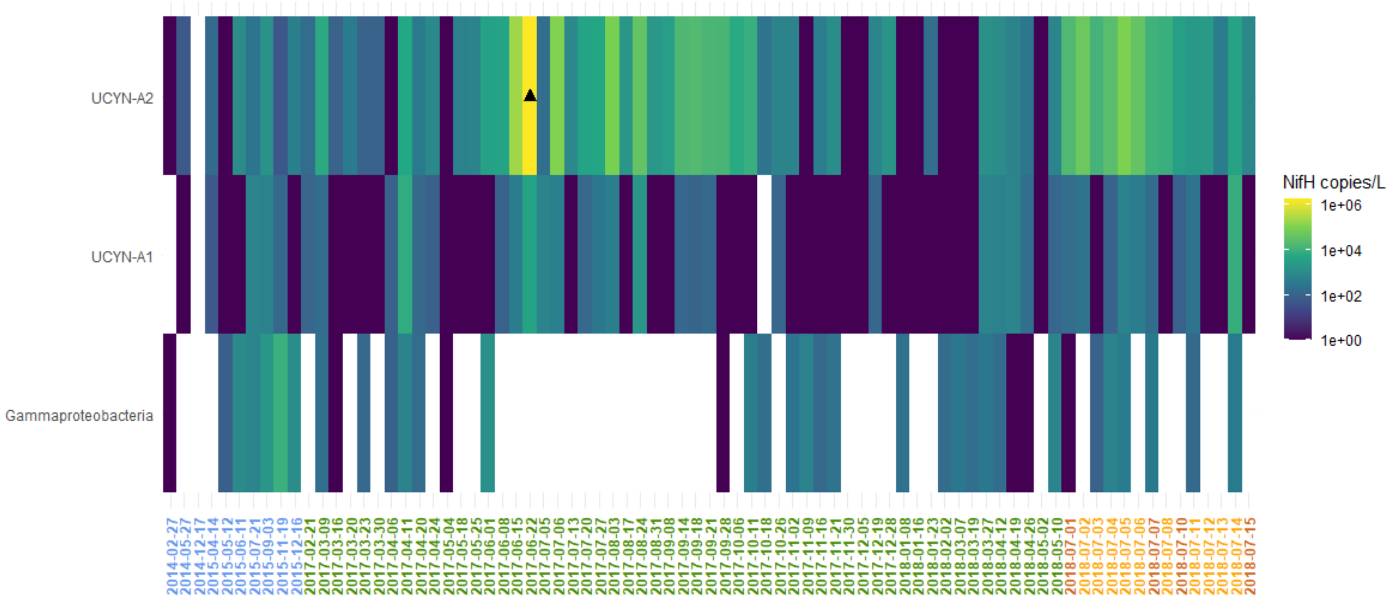

Text S1

Extended information on nutrient addition experimental setup. On 21st, February, 20th March, 4th July and 18th September 2017 surface (5 m) seawater samples were collected at a central sampling point in the Ría de Vigo (8.778º W 42.235º N) onboard R/V Kraken. Seawater was sampled in 12 l acid-cleaned Niskin bottles and filtered through a 200 µm pore size mesh to remove larger zooplankton in order to ensure good replication, given the difficulty to homogeneously sample these organisms in 12 l bottles. Subsequently, Whirl-pak® bags were gently filled with 5.4 L of sample under dim light conditions and incubated for 72 h in open tanks ashore where surface water was continuously circulated to maintain in situ temperature and light conditions. Experiments lasted 3 days and at the end of the experiments, more than a third of the incubated volume remained unsampled. Samples were taken every 24 h to monitor changes in *Chla* and PP. RNA (for *nifH* qPCR measurements) and BNF rates were sampled after 24 h incubation. The samples were analyzed using the same protocols stated in the main text for field samples. The treatments do not include Fe additions since availability of dissolved Fe is not a limiting factor in this ecosystem[6].

Table S1

Sampling summary table with the number and types of samples collected at the different sampling events. The table is also provided as an external file.

		In green color the dates when the sample exists and it was included in the manuscript dataset					
Date (dd/mm/yyyy)	Location	Hydrography (including measured dissolved inorganic nutrients)	Biological nitrogen fixation (BNF)	Nested-amplification of the *nifH* gene	nifH gene quantification through qPCR from DNA	nifH gene quantification through RT-qPCR from RNA	Nutrient addition experiment
	**TOTAL**	**75**	**34**	**71**	**78**	**15**	**4**
	**% of dates**	**95**	**43**	**90**	**99**	**19**	
27/02/2014	Shelf off Ría de A Coruña						
27/05/2014	Shelf off Ría de A Coruña						
17/12/2014	Shelf off Ría de A Coruña						
14/04/2015	Shelf off Ría de A Coruña						
12/05/2015	Shelf off Ría de A Coruña						
11/06/2015	Shelf off Ría de A Coruña						
21/07/2015	Shelf off Ría de A Coruña						
03/09/2015	Shelf off Ría de A Coruña						
19/11/2015	Shelf off Ría de A Coruña						
16/12/2015	Shelf off Ría de A Coruña						
21/02/2017	Ría de Vigo						
09/03/2017	Ría de Vigo						
16/03/2017	Ría de Vigo						
20/03/2017	Ría de Vigo						
23/03/2017	Ría de Vigo						
30/03/2017	Ría de Vigo						
06/04/2017	Ría de Vigo						
11/04/2017	Ría de Vigo						
20/04/2017	Ría de Vigo						
24/04/2017	Ría de Vigo						
04/05/2017	Ría de Vigo						
18/05/2017	Ría de Vigo						
25/05/2017	Ría de Vigo						
01/06/2017	Ría de Vigo						
08/06/2017	Ría de Vigo						
15/06/2017	Ría de Vigo						
22/06/2017	Ría de Vigo						
05/07/2017	Ría de Vigo						
06/07/2017	Ría de Vigo						
13/07/2017	Ría de Vigo						
20/07/2017	Ría de Vigo						
27/07/2017	Ría de Vigo						
03/08/2017	Ría de Vigo						
17/08/2017	Ría de Vigo						
24/08/2017	Ría de Vigo						
31/08/2017	Ría de Vigo						
08/09/2017	Ría de Vigo						
14/09/2017	Ría de Vigo						
18/09/2017	Ría de Vigo						
21/09/2017	Ría de Vigo						
28/09/2017	Ría de Vigo						
06/10/2017	Ría de Vigo						
11/10/2017	Ría de Vigo						
18/10/2017	Ría de Vigo						
26/10/2017	Ría de Vigo						
02/11/2017	Ría de Vigo						
09/11/2017	Ría de Vigo						
16/11/2017	Ría de Vigo						
21/11/2017	Ría de Vigo						
30/11/2017	Ría de Vigo						
05/12/2017	Ría de Vigo						
19/12/2017	Ría de Vigo						
28/12/2017	Ría de Vigo						
08/01/2018	Ría de Vigo						
16/01/2018	Ría de Vigo						
23/01/2018	Ría de Vigo						
02/02/2018	Ría de Vigo						
07/03/2018	Ría de Vigo						
19/03/2018	Ría de Vigo						
27/03/2018	Ría de Vigo						
12/04/2018	Ría de Vigo						
19/04/2018	Ría de Vigo						
26/04/2018	Ría de Vigo						
02/05/2018	Ría de Vigo						
10/05/2018	Ría de Vigo						
01/07/2018	Outer shelf of Ría de Pontevedra						
02/07/2018	Ría de Pontevedra						
03/07/2018	Ría de Pontevedra						
04/07/2018	Ría de Pontevedra						
05/07/2018	Ría de Pontevedra						
06/07/2018	Ría de Pontevedra						
07/07/2018	Outer shelf of Ría de Pontevedra						
08/07/2018	Ría de Pontevedra						
10/07/2018	Outer shelf of Ría de Pontevedra						
11/07/2018	Ría de Pontevedra						
12/07/2018	Ría de Pontevedra						
13/07/2018	Ría de Pontevedra						
14/07/2018	Ría de Pontevedra						
15/07/2018	Outer shelf of Ría de Pontevedra						
	TOTAL	75	34	71	78	15	4
	% of dates	95	43	90	99	19	

Table S2

Limit of detection for each one of the BNF measurements computed following[7]**, along with the BNF rate and the standard error (nmol·L^{-1}d^{-1}).** The table is also provided as an external file.

Date	Location	Limit of detection	Nitrogen fixation rate	Nitrogen fixation rate standard error
dd/mm/yyy	Surface depth	nmol N L^{-1} d^{-1}	nmol N L^{-1} d^{-1}	nmol N L^{-1} d^{-1}
27/02/2014	Shelf off Ría de A Coruña	0.07	0.05	0.003
17/12/2014	Shelf off Ría de A Coruña	0.05	0.03	0.012
14/04/2015	Shelf off Ría de A Coruña	0.13	0.10	0.024
12/05/2015	Shelf off Ría de A Coruña	0.11	0.01	0.015
11/06/2015	Shelf off Ría de A Coruña	0.05	0.04	0.025
21/07/2015	Shelf off Ría de A Coruña	0.05	0.06	0.020
03/09/2015	Shelf off Ría de A Coruña	0.06	0.08	0.011
19/11/2015	Shelf off Ría de A Coruña	0.05	0.00	0.000
16/12/2015	Shelf off Ría de A Coruña	0.04	0.00	0.000
21/02/2017	Ría de Vigo	0.21	0.02	0.000
20/03/2017	Ría de Vigo	0.26	0.11	0.000
05/07/2017	Ría de Vigo	0.13	0.43	0.202
06/07/2017	Ría de Vigo	0.13	0.30	0.117
24/08/2017	Ría de Vigo	0.14	1.42	0.290
18/09/2017	Ría de Vigo	0.17	0.59	0.222
26/10/2017	Ría de Vigo	0.08	0.09	0.040
16/11/2017	Ría de Vigo	0.06	0.02	0.009
19/12/2017	Ría de Vigo	0.06	0.03	0.006
23/01/2018	Ría de Vigo	0.05	0.02	0.008
19/04/2018	Ría de Vigo	0.19	0.01	0.008
01/07/2018	Outer shelf of Ría de Pontevedra	0.09	2.10	0.397
02/07/2018	Ría de Pontevedra	0.10	2.86	0.426
03/07/2018	Ría de Pontevedra	0.08	2.80	0.298
04/07/2018	Ría de Pontevedra	0.09	2.39	0.079
05/07/2018	Ría de Pontevedra	0.08	1.22	0.125
06/07/2018	Ría de Pontevedra	0.09	2.29	0.383
07/07/2018	Outer shelf of Ría de Pontevedra	0.13	3.17	0.367
08/07/2018	Ría de Pontevedra	0.11	0.44	0.072
10/07/2018	Outer shelf of Ría de Pontevedra	0.06	0.34	0.044
11/07/2018	Ría de Pontevedra	0.08	0.06	0.016
12/07/2018	Ría de Pontevedra	0.11	0.21	0.017
13/07/2018	Ría de Pontevedra	0.08	0.05	0.008
14/07/2018	Ría de Pontevedra	0.07	0.05	0.004
15/07/2018	Outer shelf of Ría de Pontevedra	0.05	0.13	0.052

Table S3

Primers and probes for qPCR assays. This table presents the forward primers, probes, and reverse primers used for the detection of specific taxonomic groups via qPCR, along with the corresponding references. The accession numbers or GenBank links are provided for sequence verification.

Taxonomic Group	Species	Forward primer	Probe	Reverse primer	Reference
γ-Proteobacterium	24774A11	CGGTAGAGGATCTTGA GCTTGAA	56FAM/AAGTGCTTA/ZEN/AGGTTGG CTTTGGCGACA /31ABkFQ	CAAGTGCGTGGAGTCAGGTG	[8]
UCYN-A1	http://www.ncbi.nlm.nih.gov/nuccore/AF059642	GGCTATAACAACGTTTT ATGCGTTGA	56FAM/TCCGGTGGT/ZEN/CCTGAGC CTGGA/31ABkFQ/	ACCACGACCAGCACATCCA	[9]
UCYN-A2	GenBank Accession Numbers KF806604-KF806612	GGTTACAACAACGTTTT ATGTGTTGA	FAMTCTGGTGGTCCTGAGCCCGGA-TAMRA	FAM-TCTGGTGGTCCTGAGCCCGGA-TAMRA	[10]

Table S4

Different diazotroph ASVs (n) existent in the dataset and the fraction of total reads that they represent (in percentage, %) according to the considered taxonomical groups (Class level).

Taxonomic group	ASVs (*n*)	% total reads
Deltaproteobacteria	2343	15.6
Gammaproteobacteria	1094	11.1
Epsilonproteobacteria	664	7.6
Firmicutes	607	4.2
Chlorobia	590	3.2
Bacteroidia	531	8.6
Betaproteobacteria	464	2.1
Others	444	2.4
Cyanobacteria	192	45.0
Alphaproteobacteria	101	0.16

Table S5

Results of the fit between the environmental variables and the PCoA ordination. Fit was done with the package *vegan*, function *envfit*[11]. Empirical p-values and squared correlation coefficient (r^2) for each considered variable, in decreasing order of r^2.

	p-value	r^2
Temp	0.001	0.61
%NH4	0.001	0.54
NO_3	0.001	0.45
N:P	0.001	0.44
PO_4	0.001	0.27
Salinity	0.003	0.22
PP	0.2	0.06
Chl a	0.3	0.04

Table S6

Partial weighted niche overlap (%) for each environmental factor and diazotroph group[12]**.** Bold numbers denote the existence of significant differences among niches at p<0.001 level of significance.

		Cyanobacteria	Deltaproteobacteria	Gammaproteobacteria	Epsilonproteobacteria	Bacteroidia	Firmicutes
$\%NH_4$ in DIN	Cyanobacteria	100					
	Deltaproteobacteria	**49**	100				
	Gammaproteobacteria	**30**	78	100			
	Bacteroidia	**26**	57	60	100		
	Epsilonproteobacteria	**54**	85	66	52	100	
	Firmicutes	79	68	47	74	39	100
N:P ratio	Cyanobacteria	100					
	Deltaproteobacteria	**61**	100				
	Gammaproteobacteria	**41**	71	100			
	Bacteroidia	**37**	70	68	100		
	Epsilonproteobacteria	56	82	65	79	100	
	Firmicutes	80	75	55	67	52	100
Temperature	Cyanobacteria	100					
	Deltaproteobacteria	**52**	100				
	Gammaproteobacteria	**46**	93	100			
	Bacteroidia	**34**	71	77	100		
	Epsilonproteobacteria	**49**	85	82	66	100	
	Firmicutes	6	17	17	19	16	100

Table S7

Complete ASV sequences depicted in Fig. 4C along with their nearest blast match. The table is available as an external file, here we show a screen capture of the first rows and columns.

ASV in figure 4b	Domain	Phylum	Class	Order	Family	Genus	specie	cluste	Taxa	name	blast	Sequence
Cyanobacteria UCYN-A2 (100%) - seq1	Bacteria	Cyanobacteria		Chroococcales			cyanobacte	1B	Cyanobacteria	CnbUCYNA	100	TTCCACCCGTTTAAT
Chlorobia - seq34	Bacteria	Chlorobi	Chlorobia	Chlorobiale	Chlorobiace	Chloroherp	Chloroherp	3P	Chlorobia	ChhThal2	89.908	CTCAACACGTCTTCT
Sideroxydans lithotrophicus (98%) - seq42	Bacteria	Proteobacte	Betaproteo	Gallionella	Gallionella	Sideroxydai	Sideroxydai	1P	Betaproteobacteria	SidLith2	98.165	CTCCACCCGCCTGAT
Gammaproteobacteria - seq46	Bacteria	Proteobacte	Gammapro	Pseudomor	Pseudomor	Azotobacte	Azotobacte	1G	Gammaproteobacteri	AztVin11	90.826	TTCAACGCGCTTAA
Arcobacter nitrofigilis (95%) - seq123	Bacteria	Proteobacte	Epsilonprot	Campyloba	Campyloba	Arcobacter	Arcobacter	1F	Epsilonproteobacteri	ArcNitr5	95.041	TTCTACTAGACTTAT
Epsilonproteobacteria - seq138	Bacteria	Proteobacte	Epsilonprot	Campyloba	Helicobacte	Wolinella	Wolinella	1F	Epsilonproteobacteri	WolSucc2	86.885	TTCAACAAGACTTA
Gammaproteobacteria - seq245	Bacteria	Proteobacte	Gammapro	Pseudomor	Pseudomor	Pseudomor	Pseudomor	1G	Gammaproteobacteri	PseStut3	93.578	TTCAACACGCTTAA
Firmicutes - seq280	Bacteria	Firmicutes	Clostridia	Clostridiale	Clostridiace	Alkaliphilu	Alkaliphilu	4F	Firmicutes	AlkOrem2	81.481	TTCAACTAAAAACT
Firmicutes - seq335	Bacteria	Firmicutes	Clostridia	Clostridiale	Clostridiace	Clostridium	Clostridium	4F	Firmicutes	CloSpor2	81.308	TTCTACCAGGAATT
Bacteroidia - seq379	Bacteria	Bacteroidet	Bacteroidia	Bacteroidal	Porphyromc	Paludibacte	Paludibacte	3L	Bacteroidia	PalProp2	86.239	TTCAACCAGATTAC
Gammaproteobacteria - seq402	Bacteria	Proteobacte	Gammapro	Pseudomor	Pseudomor	Azotobacte	Azotobacte	1G	Gammaproteobacteri	AztVin11	90.826	TTCAACGCGCTTAA
Desulfovibrio aespoeensis (98%) - seq427	Bacteria	Proteobacte	Deltaprote	Desulfovibı	Desulfovibı	Desulfovibı	Desulfovibı	3E	Deltaproteobacteria	DsfAesp2	98.165	CTCCACCCGTTTGCT
Chlorobia - seq483	Bacteria	Chlorobi	Chlorobia	Chlorobiale	Chlorobiace	Chloroherp	Chloroherp	3P	Chlorobia	ChhThal2	89.908	TTCAACAAGATTAC
Pelobacter carbinolicus (95%) - seq568	Bacteria	Proteobacte	Deltaprote	Desulfuron	Pelobactera	Pelobacter	Pelobacter	1A	Deltaproteobacteria	PlbCarb2	95.413	CTCAACTCGCCTGA
Deltaproteobacteria - seq616	Bacteria	Proteobacte	Deltaprote	Desulfobac	Desulfobac	Desulfatiba	Desulfatiba	3H	Deltaproteobacteria	DscAlke2	89.908	CTCCACTCGCCTGAT
Allochromatium vinosum (95%) - seq622	Bacteria	Proteobacte	Gammapro	Chromatial	Chromatiac	Allochroma	Allochroma	1G	Gammaproteobacteri	AllVino4	95.413	CTCCACCCGTCTGAT
Epsilonproteobacteria - seq665	Bacteria	Proteobacte	Epsilonprot	Campyloba	Helicobacte	Wolinella	Wolinella	1F	Epsilonproteobacteri	WolSucc2	83.471	CTCAACAAGACTTA
Chlorobia - seq689	Bacteria	Chlorobi	Chlorobia	Chlorobiale	Chlorobiace	Chloroherp	Chloroherp	3P	Chlorobia	ChhThal2	89.908	TTCAACAAGATTAT
Firmicutes - seq949	Bacteria	Firmicutes	Clostridia	Clostridiale	Clostridiace	Clostridium	Clostridium	3C	Firmicutes	CloAcet2	86.916	CTCTACAAGACTTT
Deltaproteobacteria - seq1077	Bacteria	Proteobacte	Deltaprote	Desulfobac	Desulfobul	Desulfurivi	Desulfurivi	3E	Deltaproteobacteria	DsvAlka2	88.991	CTCGACTCGCTTGC
Burkholderia sp. (99%) - seq1515	Bacteria	Proteobacte	Betaproteo	Burkholder	Burkholder	Burkholder	Burkholder	1K	Betaproteobacteria	BurSp151	99.074	CTCAACGCGTCTTA
Cyanobacteria UCYN-A2 (100%) - seq1	Bacteria	Cyanobacteria		Chroococcales			cyanobacte	1B	Cyanobacteria	CnbUCYNA	100	TTCCACCCGTTTAAT
Methylobacter tundripaludum (100%) - seq	Bacteria	Proteobacte	Gammapro	Methylococ	Methylococ	Methylobac	Methylobac	1O	Gammaproteobacteri	MtbTund4	100	TTCTACCCGTTTAAT
Epsilonproteobacteria - seq75	Bacteria	Proteobacte	Epsilonprot	Campyloba	Campyloba	Arcobacter	Arcobacter	1F	Epsilonproteobacteri	ArcNitr5	93.388	CTCAACAAGATTGA
Pelobacter carbinolicus (95%) - seq80	Bacteria	Proteobacte	Deltaprote	Desulfuron	Pelobactera	Pelobacter	Pelobacter	1A	Deltaproteobacteria	PlbCarb2	95.413	CTCGACTCGTCTGA
Firmicutes - seq255	Bacteria	Firmicutes	Clostridia	Clostridiale	Clostridiace	Clostridium	Clostridium	3C	Firmicutes	CloLjun5	91.589	TTCAACAAGATTGA
Gammaproteobacteria - seq292	Bacteria	Proteobacte	Gammapro	Pseudomor	Pseudomor	Pseudomor	Pseudomor	1G	Gammaproteobacteri	PseStut3	93.578	TTCAACTCGTCTAAT
Deltaproteobacteria - seq296	Bacteria	Proteobacte	Deltaprote	Desulfovibı	Desulfovibı	Desulfovibı	Desulfovibı	3P	Deltaproteobacteria	DsfMagn2	89.908	TTCAACAAGATTAC
Candidatus Azobacteroides (95%) - seq340	Bacteria	Bacteroidet	Bacteroidia	Bacteroidal	Candidatus	Azobacteroi	Candidatus	3L	Bacteroidia	CanAzob2	95.413	TTCCACTCGTTTACT
Chlorobia - seq349	Bacteria	Chlorobi	Chlorobia	Chlorobiale	Chlorobiace	Chloroherp	Chloroherp	3P	Chlorobia	ChhThal2	85.321	TTCAACACGATTAT
Deltaproteobacteria - seq353	Bacteria	Proteobacte	Deltaprote	Desulfovibı	Desulfovibı	Desulfovibı	Desulfovibı	3E	Deltaproteobacteria	DsfAesp2	93.578	CTCAACCAGATTAT
Bacteroidia - seq391	Bacteria	Bacteroidet	Bacteroidia	Bacteroidal	Porphyromc	Paludibacte	Paludibacte	3L	Bacteroidia	PalProp2	87.156	TTCAACAAGATTAT
Deltaproteobacteria - seq447	Bacteria	Proteobacte	Deltaprote	Desulfovibı	Desulfovibı	Desulfovibı	Desulfovibı	3E	Deltaproteobacteria	DsfAesp2	94.495	CTCAACCAGATTAT
Desulfurivibrio alkaliphilus (95%) - seq601	Bacteria	Proteobacte	Deltaprote	Desulfobac	Desulfobul	Desulfurivi	Desulfurivi	3E	Deltaproteobacteria	DsvAlka2	95.413	TTCAACGAGGCTTT
Firmicutes - seq710	Bacteria	Firmicutes	Clostridia	Clostridiale	Clostridiace	Clostridium	Clostridium	3C	Firmicutes	CloCel11	93.458	TTCAACTCGTCTGC
Firmicutes - seq792	Bacteria	Firmicutes	Clostridia	Clostridiale	Clostridiace	Clostridium	Clostridium	3C	Firmicutes	CloCel11	89.72	TTCGACAAGACTAA
Firmicutes - seq811	Bacteria	Firmicutes	Clostridia	Clostridiale	Clostridiace	Clostridium	Clostridium	3C	Firmicutes	CloKluy8	87.85	CTCCACACGTTTGG
Burkholderia xenovorans (99%) - seq1180	Bacteria	Proteobacte	Betaproteo	Burkholder	Burkholder	Burkholder	Burkholder	1K	Betaproteobacteria	BurXeno2	99.074	CTCGACGCGCCTGA
Gammaproteobacteria - seq2124	Bacteria	Proteobacte	Gammapro	Enterobacte	Enterobacte	Klebsiella	Klebsiella	1G	Gammaproteobacteri	KleSpe36	89.908	CTCAACCCGTCTTAT

References

1. Shao Z, Xu Y, Wang H, Luo W, Wang L, Huang Y, et al. Global oceanic diazotroph database version 2 and elevated estimate of global oceanic N_2 fixation. *Earth System Science Data* 2023; **15**: 3673–3709.

2. Harding KJ, Turk-Kubo KA, Mak EWK, Weber PK, Mayali X, Zehr JP. Cell-specific measurements show nitrogen fixation by particle-attached putative non-cyanobacterial diazotrophs in the North Pacific Subtropical Gyre. *Nat Commun* 2022; **13**: 6979.

3. Krupke A, Musat N, LaRoche J, Mohr W, Fuchs BM, Amann RI, et al. In situ identification and N2 and C fixation rates of uncultivated cyanobacteria populations. *Systematic and Applied Microbiology* 2013; **36**: 259–271.

4. Shao Z, Luo Y-W. Controlling factors on the global distribution of a representative marine non-cyanobacterial diazotroph phylotype (Gamma A). *Biogeosciences* 2022; **19**: 2939–2952.

5. Comesaña A, Fernández-Castro B, Chouciño P, Fernández E, Fuentes-Lema A, Gilcoto M, et al. Mixing and Phytoplankton Growth in an Upwelling System. *Frontiers in Marine Science* 2021; **8**.

6. Filgueiras AV, Prego R. Biogeochemical Fluxes of Iron from Rainwater, Rivers and Sewage to a Galician Ria (NW Iberian Peninsula). Natural versus Anthropogenic Contributions. *Biogeochemistry* 2007; **86**: 319–329.

7. White AE, Granger J, Selden C, Gradoville MR, Potts L, Bourbonnais A, et al. A critical review of the 15N2 tracer method to measure diazotrophic production in pelagic ecosystems. *Limnology and Oceanography: Methods* 2020; **18**: 129–147.

8. Moisander PH, Beinart RA, Voss M, Zehr JP. Diversity and abundance of diazotrophic microorganisms in the South China Sea during intermonsoon. *ISME J* 2008; **2**: 954–967.

9. Church MJ, Short CM, Jenkins BD, Karl DM, Zehr JP. Temporal Patterns of Nitrogenase Gene (nifH) Expression in the Oligotrophic North Pacific Ocean. *Applied and Environmental Microbiology* 2005; **71**: 5362–5370.

10. Thompson A, Carter BJ, Turk-Kubo K, Malfatti F, Azam F, Zehr JP. Genetic diversity of the unicellular nitrogen-fixing cyanobacteria UCYN-A and its prymnesiophyte host. *Environmental Microbiology* 2014; **16**: 3238–3249.

11. Oksanen J, Simpson GL, Blanchet FG, Kindt R, Legendre P, Minchin PR, et al. vegan: Community Ecology Package. 2024.

12. Geange SW, Pledger S, Burns KC, Shima JS. A unified analysis of niche overlap incorporating data of different types. *Methods in Ecology and Evolution* 2011; **2**: 175–184.
